# Supplementary material for: The effect of excessive trabeculation on cardiac rotation—A multimodal imaging study
Source: PLoS One. 2024 Sep 5;19(9):e0308035. doi: 10.1371/journal.pone.0308035 (PMC11376564; doi:10.1371/journal.pone.0308035)
Supplement: S2 Table — ICC = intraclass correlation: average measures (95% confidential interval lower and upper band), CMR = cardiac magnetic resonance imaging, LVEDVi = left ventricular end-diastolic volume index, LVESVi = left ventricular end-systolic volume index, LVSVi = left ventricular stroke volume index, LVEF = left ventricular ejection fraction, LVTMi = left ventricular total mass index, LVTPMi = left ventricular trabeculated and papillary muscle mass index, NA = not applicable. (DOCX) [file pone.0308035.s002.docx]

**S2 Table**  Interobserver agreement (ICC) for the measured functional and rotational parameters with both CMR and echocardiography

|  | **Intraclass correlation coefficient** | |
| --- | --- | --- |
|  | **CMR** | **Echocardiography** |
| **LVEDVi** | 0.996 (0.989-0.999) | 0.937 (0.814-0.979) |
| **LVESVi** | 0.984 (0.952-0.995) | 0.958 (0.874-0.986) |
| **LVSVi** | 0.970 (0.912-0.990) | 0.896 (0.689-0.965) |
| **LVEF** | 0.953 (0.861-0.984) | 0.839 (0.519-0.946) |
| **LVTMi** | 0.996 (0.987-0.998) | NA |
| **LVTPMi** | 0.963 (0.892-0.987) | NA |
| **Basal rotation (°)** | 0.849 (0.608-0.947) | 0.680 (0.470-0.893) |
| **Apical rotation (°)** | 0.797 (0.394-0.932) | 0.885 (0.658-0.961) |
| **Net cardiac twist (°)** | 0.844 (0.536-0.948) | 0.701 (0.108-0.899) |

ICC = intraclass correlation: average measures (95% confidential interval lower and upper band), CMR = cardiac magnetic resonance imaging, LVEDVi = left ventricular end-diastolic volume index, LVESVi = left ventricular end-systolic volume index, LVSVi = left ventricular stroke volume index, LVEF = left ventricular ejection fraction, LVTMi = left ventricular total mass index, LVTPMi = left ventricular trabeculated and papillary muscle mass index, NA = not applicable
